# The association of social isolation and loneliness with sarcopenia among the middle-aged and elderly in China

**DOI:** 10.1186/s12888-024-05958-y

**Published:** 2024-07-18

**Authors:** Lei Tu, Yiqun Li, Xiao Ren, Minglan Jiang, Longyang Han, Xiaowei Zheng

**Affiliations:** Public Health Research Center, Department of Public Health and Preventive Medicine, Wuxi School of Medicine, Jiangnan University, 1800 Lihu Road, Binhu District, Wuxi, Jiangsu Province 214122 China

**Keywords:** Social isolation, Loneliness, Sarcopenia, Cross-sectional study, Longitudinal study

## Abstract

**Objectives:**

This study examined the relationship of social isolation and loneliness on sarcopenia among Chinese middle-aged and elderly people.

**Methods:**

Social isolation, loneliness, and sarcopenia were measured at baseline. Follow-up measures of new-onset sarcopenia were obtained 4 years later. Then used logistic regression to evaluate the association between social isolation, loneliness and sarcopenia.

**Results:**

In cross-sectional analysis, social isolation and loneliness are significantly associated with sarcopenia [OR = 1.88 (95% CI = 1.54–2.28)]. In longitudinal analysis, social isolation and loneliness are significantly associated with sarcopenia [OR = 1.09 (95% CI = 0.71–1.69)]. Social isolation and loneliness have a synergistic effect. Among them, individuals over 60 years old [OR = 2.01 (95% CI = 1.37–2.96)] and those without social support [OR = 2.64 (1.61–4.32), *P*-for interaction < 0.001] are at higher risk.

**Conclusion:**

Social isolation and loneliness were significantly associated with sarcopenia, and there was a synergistic effect between social isolation and loneliness.

**Supplementary Information:**

The online version contains supplementary material available at 10.1186/s12888-024-05958-y.

## Introduction

China entered the aging society as early as 2000, and the rate of population aging is very alarming [1, 2]. Comparing the data from the seventh national census in 2020 and the fifth national census in 2000, the population aged 60 and above in China has increased by over 102 million in 20 years, representing a growth rate of 115.96% [1, 2]. As the signs of aging become increasingly apparent in China, the concept of “healthy aging” has been introduced to our country. This has led to a growing concern for the health of the middle-aged and elderly population. As a geriatric syndrome, sarcopenia has gradually attracted attention. The Asian Working Group for Sarcopenia (AWGS) reached a consensus in 2014 to define sarcopenia as “the age-related loss of muscle mass accompanied by low muscle strength and/or low physical performance” [3]. According to the latest criteria of AWGS 2019, a recent meta-analysis on the prevalence of sarcopenia in Chinese older adults showed that the overall prevalence of sarcopenia in older adults in China was 14% (95% CI = 11-18%) [4]. In Comparison to Southeast Asian countries, East Asian countries like China, Japan, and South Korea have a higher prevalence of sarcopenia [5]. Sarcopenia is a progressive and generalized skeletal muscle disorder that may be associated with adverse outcomes, such as falls, fractures, disability, and death [3]. One of the important reasons for the occurrence of sarcopenia is the change in baseline strength and muscle size in the body due to increasing age [6]. However, the aging of the population is an unavoidable problem in the country at present. Therefore, the purpose of studying the factors related to the development of sarcopenia is to intervene in a timely manner and reduce the economic burden of health care in the country and develop the promotion of healthy aging.

Several studies have shown that aging, malnutrition, lack of physical activity, other chronic non-communicable diseases (such as: diabetes mellitus, cardiovascular disease), and inflammation increase the risk of sarcopenia [7, 8]. In terms of current research on the risk factors associated with the development of sarcopenia, most studies have focused on the diet, lifestyle habits, and the presence of common chronic noncommunicable diseases in patients with sarcopenia. However, fewer studies have focused on social factors (social isolation) and psychological factors (loneliness) in middle-aged and older adults. Some scholars believe that social isolation and loneliness are the same concept, which is not the case. Social isolation is a negative emotion resulting from lack of meaningful relationships and social integration. It is measured objectively by the absence of a large network, diverse network connections, and frequent exposure to social networks. On the other hand, loneliness is a subjective feeling of perceiving a lack of companionship or the loss of someone to rely on [9, 10]. Both objective social isolation and subjective loneliness can lead to an increased risk of death in older adults, which is even higher compared to other recognized risk factors for death (frailty) [11]. Social isolation and loneliness are now recognized as public health issues and challenges [12, 13].

Few studies have focused on the association between social isolation and loneliness with sarcopenia. One of the indicators of muscle mass measured by AWGS is grip strength, and in a longitudinal study, it was found that social isolation showed a positive correlation with decreased grip strength in men, and decreased grip strength showed a high level of correlation with loneliness in women [14]. It has also been shown that living alone may increase loneliness in middle-aged and older adults, and that loneliness is associated with decreased motor function, malnutrition, systemic arterial hypertension, and frailty, which in turn increase social isolation [15, 16], all of which increase the risk of sarcopenia [15].

Therefore, this study will explore and discuss the association between social isolation and loneliness with sarcopenia.

## Methods

### Sample

Data from the China Health and Retirement Longitudinal Study (CHARLS), a nationally representative longitudinal study that collects high-quality microdata on households and individuals of adults aged 45 and above. CHARLS was conducted in 2011, covering multiple counties and villages, and the sample has been followed up every two to three years since then.

**Fig. 1:**
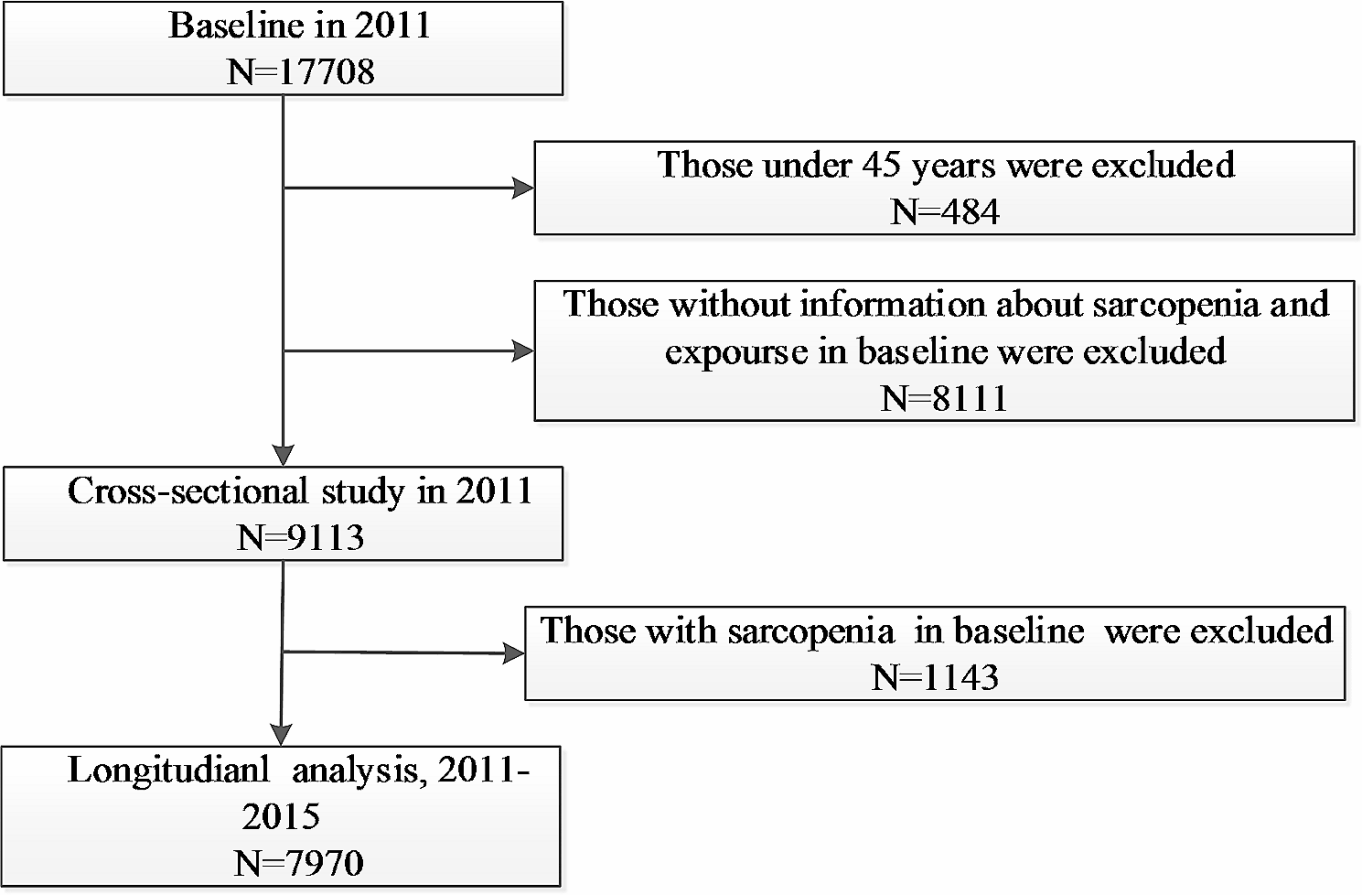
Flow chart of sample selection and the exclusion criteria

In this study, we retrospectively analyzed data from CHARLS 2011 and 2015. The study was divided into two parts: (1) In the cross-sectional analysis, we used data from the CHARLS 2011. A total of 17,708 participants were included, of which 8,595 were excluded due to lack of information related to sarcopenia, baseline exposure data (*n* = 8,111), and age less than 45 years (*n* = 484), leaving 9,113 participants for the cross-sectional study. (2) In longitudinal analyses, we further excluded 1,143 participants with sarcopenia in CHARLS 2011. Finally, 7,970 participants in CHARLS 2011–2015 were included in the longitudinal analysis. The detailed selection process is shown in Fig. 1.

### Assessment of sarcopenia status

This study utilized the AWGS 2019 recommended diagnostic algorithm to measure muscle strength, physical function, and limb skeletal muscle mass to define sarcopenia prevalence and incidence in both cross-sectional analysis and longitudinal analyses [3]. Participants who did not show any low muscle mass, low skeletal muscle mass or low physical functioning were recognized as having “no sarcopenia”. “possible sarcopenia” is defined as either low muscle strength or low physical performance only. Sarcopenia is diagnosed when low muscle mass plus low muscle strength or low physical performance. When low muscle strength, low muscle mass and low physical performance are all detected, severe sarcopenia will be considered. We attribute possible sarcopenia, sarcopenia and severe sarcopenia to patients with sarcopenia. And the outcome for cross-sectional analysis is defined as sarcopenia at baseline, and the outcome for longitudinal analysis is defined as sarcopenia at a four-year follow-up in patients who did not have sarcopenia at baseline.

### Assessment of loneliness and social isolation

In the current study, loneliness was measured using an item benchmark from the Center for Epidemiological Studies Depression Scale (CESD), “In the past week, how often have you felt loneliness? " It is categorized into four responses from “never” to “always“ [17, 18]. This single-item measure is highly correlated with multiple loneliness scales and has been used extensively in many previous studies. Loneliness was categorized into two categories [0 (not loneliness) = rarely or never loneliness, 1 (loneliness) = sometimes, occasionally, or most of the time] [17, 18].

Indicators of social isolation were derived from three items at baseline [19, 20]. Points were assigned based on the following baseline conditions of the participant, with one condition assigned a value of 1. The participant was unmarried (unmarried, separated, divorced, widowed) or living alone; had contact with children or family members less than once a week (meeting, phone call, e-mail); and had not participated in any social activities in the past month (sports or social activities, playing mahjong or cards, interacting with friends, charity or volunteer work, community activities). Scores range from 0 to 3, with higher scores indicating higher social isolation.

### Covariates

The covariates were collected at baseline including age, sex, place of residence (rural vs. urban), educational level (illiteracy; primary school; middle school; high school or above), drinking status (ever drinking vs. never drinking), smoking status (ever smoking vs. never smoking), systolic blood pressure, the presence or absence of other chronic diseases (dyslipidemia, diabetes mellitus, cancer, chronic lung disease, stroke, liver disease, arthritis, digestive disease) and medications (anti-hypertensive, anti-dyslipidemic and anti-diabetic). “Ever smoking” means that the respondent reported smoking at some point, and “never smoking” means that the respondent reported never having smoked. “Ever drinking” means that the respondent reports having had an alcoholic beverage in the past, and “never drinking” means that the respondent reported not having any alcoholic beverage in the past. Blood pressure was measured with an electronic sphygmomanometer (Omron HEM-7200 Monitor) after 5 min of rest in the sitting position and was defined as the average of three separate measurements. Hypertension was defined as systolic blood pressure ≥ 140 mm Hg, diastolic blood pressure ≥ 90 mm Hg, current use of antihypertensive medications, or self-reported history of hypertension. Moreover, dyslipidemia was defined as triglycerides ≥ 150 mg/dL, or total cholesterol ≥ 240 mg/dL, or high-density lipoprotein cholesterol < 40 mg/dL, or low-density lipoproteins cholesterol ≥ 160 mg/dL, or current use of the lipid-lowering medications, or self-reported history of dyslipidemia. And diabetes was defined as fasting glucose ≥ 126 mg/dL, or glycosylated hemoglobin (HbA1c) ≥ 6.5%, or treatment for diabetes mellitus, or self-reported history of diabetes. Social support was measured by three dimensions: family size, proximity of support, and social involvement according to previous study [21].

### Statistical analysis

For baseline information characteristics, continuous data were described using means and standard deviations, and categorical data were described using percentages. The two independent samples t-test and χ^2^ test were used to examine the differences in characteristics between participants with or without sarcopenia.

In cross-sectional and longitudinal analyses, multivariable logistic regression models were applied to calculate the odds ratio (OR) and 95% confidence interval (95% CI) between social isolation and loneliness with sarcopenia. Three models were fitted to the analysis. Model 1 was not adjusted for any factors, model 2 was adjusted for age and sex, and model 3 was adjusted for age, sex, place of residence, education level, smoking, drinking, systolic blood pressure, history of chronic diseases (dyslipidemia, diabetes mellitus, cancer, chronic lung disease, stroke, liver disease, arthritis, digestive disease) and use of medications (anti-hypertensive, anti-dyslipidemic and anti-diabetic).

In the longitudinal analysis, subgroup analyses were performed to evaluate the association between social isolation and loneliness with sarcopenia according to place of residence, social support, place of residence, age and sex, and the multiplicative interactions was calculated between social isolation, loneliness and subgroups [22]. Furthermore, we merged those with possible sarcopenia into sarcopenia group in sensitivity analyses to test the robustness of our findings.

All analyses were conducted using SAS version 6.0 (SAS Institute, Inc., Cary, NC) statistical software.

## Results

### Characteristics of participants’ baseline data

A total of 9,113 participants were included in this study, with an average age of 58.61 (± 8.91) years. According to the baseline data of CHARLS in 2011 and the definition of sarcopenia by AWGS in 2019, there were a total of 7,970 participants without sarcopenia and 1,143 participants with sarcopenia. Comparison of baseline data between the two groups revealed significant differences in age, sex, place of residence, education level, history of dyslipidemia and diabetes mellitus, smoking, drinking, body mass index, and diastolic blood pressure (*P-*for interaction < 0.005;Table 1).

**Table 1:** Baseline characteristics of the study participants with or without Sarcopenia in cross-sectional study

Characteristics	Total sample	Sarcopenia		*P* value
		Without	With	
No. of subjects	9113	7970	1143	
Age, years	58.61 ± 8.91	57.55 ± 8.36	66.01 ± 9.07	< 0.001
Sex, n (%)				< 0.001
Male	4273(46.89)	3886(48.76)	387(33.86)	
Female	4840(53.11)	4084(51.24)	756(66.14)	
Place of residence, n (%)				< 0.001
Urban	3018(33.12)	2752(34.53)	266(23.27)	
Rural	6095(66.88)	5218(65.47)	877(76.73)	
Education level, n (%)				< 0.001
Below primary school	2578(28.29)	2021(25.36)	557(48.73)	
Primary school	3804(41.74)	3342(41.93)	462(40.42)	
Middle school	1866(20.48)	1775(22.27)	91(7.96)	
High school or above	832(10.44)	832(10.44)	33(2.89)	
Chronic diseases history				
Hypertension, n (%)	1957(21.473)	1724(21.63)	233(20.38)	0.355
Dyslipidemia, n (%)	854(9.37)	809(10.15)	45(3.94)	< 0.001
Diabetes mellitus, n (%)	598(6.56)	544(6.83)	54(4.72)	0.007
Smoking, n (%)	3585(39.34)	3193(40.06)	392(34.30)	< 0.001
Drinking, n (%)	3500(38.41)	3145(39.46)	355(31.06)	< 0.001
BMI, kg/m^2^	23.15(20.87–25.80)	23.70(21.56–26.20)	19.64(18.41–20.91)	< 0.001
SBP, mmHg	129.41 ± 20.18	129.37 ± 19.79	129.70 ± 22.22	0.608
DBP, mmHg	75.45 ± 11.68	75.90 ± 11.60	72.33 ± 11.80	< 0.001

BMI: body mass index; SBP: systolic blood pressure; DBP: diastolic blood pressure; Continuous variables are expressed as mean ± standard deviation, or as median (interquartile range). Categorical variables are expressed as frequency (percent)

### Association between social isolation and loneliness on sarcopenia prevalence in cross-sectional study

In the baseline data, the prevalence of sarcopenia is significantly higher in the population experiencing both social isolation and loneliness compared to those experiencing only social isolation or loneliness (20.84% vs. 15.54% vs. 11.99%; Table 2). After adjusting for all covariates in the regression analysis, individuals with social isolation or loneliness were associated with a higher risk of sarcopenia, with corresponding OR (95%CI) was 1.33(1.05–1.72) and 1.51(1.31–1.74), respectively (Table 2). Furthermore, we found significant multiplicative interactions of social isolation and loneliness on sarcopenia with sarcopenia risk. Compared those without both social isolation and loneliness, individuals with social isolation alone (OR = 1.53, 95%CI 1.21–1.94), or loneliness alone (OR = 1.62, 95%CI 1.36–1.93), or with both social isolation and loneliness (OR = 1.88, 95%CI 1.54–2.28) were associated with increased risk of sarcopenia (Table 2).

**Table 2:** Association between social isolation and loneliness on Sarcopenia prevalence in cross-sectional study (*N* = 9,113)

Variable	Case, *n* (%)	Model 1	Model 2	Model 3
**Social isolation**				
No	695(10.69)	1.00(Ref)	1.00(Ref)	1.00(Ref)
Yes	448(17.15)	1.73(1.52–1.96)	1.43(1.25–1.65)	1.33(1.05–1.72)
**Loneliness**				
No	385(8.10)	1.00(Ref)	1.00(Ref)	1.00(Ref)
Yes	758(17.39)	2.39(2.10–2.72)	1.66(1.44–1.91)	1.51(1.31–1.74)
**Combined effect of social isolation and loneliness**				
Neither social isolation or loneliness	254(6.94)	1.00(Ref)	1.00(Ref)	1.00(Ref)
Social isolation alone	131(11.99)	1.83(1.46–2.28)	1.65(1.31–2.09)	1.53(1.21–1.94)
Loneliness alone	441(15.54)	2.47(2.10–2.91)	1.78(1.50–2.11)	1.62(1.36–1.93)
Both social isolation and loneliness	317(20.84)	3.53(2.96–4.22)	2.16(1.78–2.61)	1.88(1.54–2.28)

Model 1 was unadjusted

Model 2 was adjusted for age and sex

Model 3 was adjusted for adjusted for age, sex, place of residence, education level, smoking, drinking, systolic blood pressure, history of chronic diseases and medications

### Longitudinal association between social isolation and loneliness on sarcopenia incidence

After 4 years of follow-up, a total of 305 participants were diagnosed with sarcopenia (Table 3). Participants with social isolation or loneliness were associated with an increased risk of sarcopenia incidence, with corresponding OR (95%CI) was 1.34(1.05–1.72) and 1.31(1.02–1.69), respectively (Table 3). After further adjusting for all covariates, compared those without both social isolation and loneliness, those with social isolation alone were not associated with sarcopenia incidence (OR = 1.09, 95%CI 0.71–1.69). While those with loneliness alone (OR = 1.27, 95%CI 1.05–1.59), or with both social isolation and loneliness (OR = 1.67, 95%CI 1.20–2.32) were associated with increased risk of sarcopenia (Table 3).

**Table 3:** Longitudinal association between social isolation and loneliness on sarcopenia incidence (*N* = 7,970)

Variable	Case, *n* (%)	Model 1	Model 2	Model 3
**Social isolation**				
No	187(3.22)	1.00(Ref)	1.00(Ref)	1.00(Ref)
Yes	118(5.45)	1.73(1.37–2.19)	1.46(1.15–1.87)	1.34(1.05–1.72)
**Loneliness**				
No	112(2.56)	1.00(Ref)	1.00(Ref)	1.00(Ref)
Yes	193(5.36)	2.15(1.70–2.73)	1.50(1.17–1.92)	1.31(1.02–1.69)
**Combined effect of social isolation and loneliness**				
Neither social isolation or loneliness	82(2.41)	1.00(Ref)	1.00(Ref)	1.00(Ref)
Social isolation alone	30(3.12)	1.31(0.85-2.00)	1.22(0.79–1.88)	1.09(0.71–1.69)
Loneliness alone	105(4.38)	1.86(1.39–2.49)	1.34(1.09–1.82)	1.27(1.05–1.59)
Both social isolation and loneliness	88(7.313)	3.20(2.35–4.35)	2.00(1.45–2.77)	1.67(1.20–2.32)

Model 1 was unadjusted

Model 2 was adjusted for age and sex

Model 3 was adjusted for adjusted for age, sex, place of residence, education level, smoking, drinking, systolic blood pressure, history of chronic diseases and medications

### Stratified and sensitivity analysis of longitudinal associations between social isolation and loneliness with the onset of sarcopenia

Firstly, the analysis by subgroups of residence (Table S1) showed that only loneliness was statistically different between rural and urban areas (*P-*for interaction = 0.019). However, there is no statistical difference between social isolation and loneliness based on the place of residence (rural/urban), indicating that the place of residence does not affect the relationship between social isolation and loneliness with sarcopenia (Table S1). The social isolation and loneliness show significant differences among different age groups (*P-*for interaction < 0.001, Table S2). Social isolation alone and loneliness alone were not significantly associated with sarcopenia in the group less than 60 years old. However, in the group aged 60 years and above, there was a significant association between the combined effect of social isolation and loneliness with sarcopenia incidence (OR = 2.01, 95% CI 1.37–2.96) (Table S2). When it comes to gender subgroups, there is no statistically significant difference in social isolation and loneliness between males and females (Table S3). Table S4 shows there was no significant association between social isolation and loneliness with sarcopenia in individuals with social support (OR = 1.37, 95% CI 0.88–2.14, *P* = 0.169); However, there was a significant correlation between social isolation and loneliness with sarcopenia in individuals without social support (OR = 2.64, 95% CI 1.61–4.32, *P-*for interaction < 0.001) (Table S4).

In the sensitivity analysis, participants diagnosed with probable sarcopenia were included in the group of sarcopenia, and sensitivity analyses showed a significant association between social isolation and loneliness and sarcopenia after adjusting for all covariates. This is consistent with the longitudinal association analysis between social isolation and loneliness and sarcopenia described above (Table S5). Those with loneliness alone (OR = 1.32, 95%CI 1.10–1.57), or with both social isolation and loneliness (OR = 1.51, 95%CI 1.25–1.82) were associated with increased risk of sarcopenia (Table S5).

## Discussion

In this study, we initially utilized the baseline data from CHARLS 2011 for cross-sectional analysis. Our findings revealed that both social isolation alone and loneliness alone were significantly associated with sarcopenia. This result is consistent with the findings of Lin, Y. H. et al. and Pegorari, M. S. et al [15, 23]. Moreover, a synergistic effect between social isolation and loneliness has been identified as a high-risk factor for sarcopenia. Subsequently, a longitudinal analysis of the collected cohort data revealed that while social isolation alone was not significantly related to sarcopenia, loneliness alone was significantly associated with sarcopenia. The interaction between social isolation and loneliness also demonstrated a significant association with sarcopenia.

The causes of sarcopenia are diverse. Malnutrition, physical inactivity, age-related decreased hormone concentrations, and inflammation are all risk factors for sarcopenia [24, 25]. Sarcopenia is also linked to other underlying diseases, such as cachexia, sarcopenic obesity, and frailty [26]. Frailty has been identified as one of the recognized risk factors for mortality [11]. There is a significant correlation between frailty and sarcopenia, with the majority of frail older people experiencing sarcopenia [26]. As we all know, social isolation and loneliness are significant factors that affect people’s psychology, and many studies have pointed out that social loneliness and loneliness are related to the onset of depression [10, 16].However, Hermes GL et al. discovered that social loneliness and loneliness not only affect people’s psychological well-being but also affect the physical activity of the human body. Specifically, individuals who have experienced long-term social loneliness and loneliness are at increased risk of inflammatory diseases [24, 27], which provides a physiological basis for the prevalence of sarcopenia [28]. Loneliness and frailty are interrelated, and shifts in early loneliness can create a vicious cycle that contributes to early weakness in the individuals, leading to a gradual decrease in physical activity and social interaction, ultimately resulting in late-stage loneliness [28]. Both social loneliness and loneliness have an impact on the grip strength of older Chinese adults, and the higher the social loneliness and loneliness, the faster the grip strength declines [14]. In Shimamoto, J.‘s the social-ecological model, he attributes social isolation to interpersonal factors related to sarcopenia [29]. Many scholars have conducted surveys on the elderly during COVID-19 [15, 30], and most individuals experienced social isolation and profound loneliness during this period [31]. They found that due to the elderly being in isolation during COVID-19, coupled with poor social interaction and a sudden decrease in physical activity, it led to muscle atrophy in the elderly, and even the development of sarcopenia [15, 30]. In their research, social isolation or loneliness was identified as risk factors for sarcopenia. However, the results of our study show that in longitudinal studies, after adjusting for all covariates, compared those without both social isolation and loneliness, those with social isolation alone were not associated with sarcopenia. This contradicts the research conducted by scholars in other countries. However, in China, the general public still lacks awareness of social isolation. In fact, most research results on the negative impacts of social isolation are generated in Western countries, and may not be fully applicable to the situation in China [32]. Additionally, in this longitudinal study, the proportion of people who experience social isolation alone is only 3.12%, which may lead to selection bias due to the small number of individuals experiencing social isolation alone. Furthermore, we speculate that elderly individuals experiencing social isolation are less likely to engage in outdoor activities or participate in collective community activities, the number of individuals available for investigation is already limited. Additionally, the presence of a certain degree of loss to follow-up over the 4-year follow-up period in this longitudinal study may have led to inconsistencies in our results compared to others. All in all, combined with our study, we believe that social isolation and loneliness are associated with a high level of sarcopenia due to the synergistic effect of the interaction between social isolation and loneliness. However, the mechanism behind the synergistic effect of social isolation and loneliness on sarcopenia needs further study. In other words, we are not yet clear about the percentage of the respective effects of social isolation and loneliness on sarcopenia under the synergistic effect.

In a study of the elderly population in the United States, Henning-Smith C et al. found that rural residents, in comparison to urban residents, had a greater number of children and grandchildren, as well as more friends. Residents who had timely support from friends and had more children showed lower levels of loneliness, while those who engaged in excessive community activities showed a high level of loneliness [33]. Therefore, even when some groups maintain frequent social contacts and community engagement, it does not necessarily mean that they do not subjectively feel lonely [34]. Individuals may experience social isolation and loneliness simultaneously.

Interestingly, in this study, unlike other analyses, we conducted separate regression analyses for groups experiencing only social isolation and only loneliness in relation to sarcopenia. If we had not separated individuals who experience both social isolation and loneliness (i.e., those who experience both) for analysis, our results would not have been consistent with the longitudinal analysis conducted by Giné-Garriga M et al. Prior to investigating the interaction between social isolation and loneliness, our research indicated a weak correlation between loneliness and sarcopenia. However, Giné-Garriga M et al.‘s study suggested that there was no significant association between loneliness and sarcopenia. This discrepancy may be attributed to variations in data sources, different follow-up periods, and a lack of long-term studies on the association between loneliness and sarcopenia. Giné-Garriga M et al.‘s study examined loneliness using data from the European SHARE project, which involved participants aged 60 and above. That study only conducted a 2-year follow-up [13].

Sarcopenia is a recognized geriatric syndrome, and in our study, we found that groups higher than 60 years of age showed social isolation and loneliness to be associated with sarcopenia, whereas social isolation and loneliness were not significantly associated with sarcopenia in groups between 45 and 60 years old. We also found that social isolation and loneliness were statistically different in both different age groups, suggesting that social isolation and loneliness still occurs predominantly in the older. The focus of our attention needs to remain on the older.

Social isolation and loneliness are multidisciplinary and multidimensional concepts that are also linked to other concepts such as social support [28]. Social support is a factor that is not only effective in alleviating loneliness [35], but it also plays a key role in the social connection and physical and mental health of the population [36]. The likelihood of social isolation and loneliness is higher in populations with lower levels of social support, and for this reason we conducted a subgroup analysis for social support. We found no significant association between social isolation and loneliness with sarcopenia for the group with social support; however, for the group without social support, social isolation and loneliness were significantly associated with sarcopenia. The quality of social support is more important than the number of social contacts for the physical and mental health of the older [37]. Many studies have found that social support is associated with depression, cardiovascular disease, and diabetes [36, 38, 39]. Therefore, when we intervene on physical actions and mental activities for the middle-aged and elderly in China, not only do we need to target social isolation and loneliness-related aspects for improvement, but we also need to adjust interventions on the quality of social support-related components.

It is worth noting that we found no statistical differences in social isolation and loneliness among different types of places of residence and genders. Therefore, we believe that in subgroup analyses related to place of residence and gender, the association between social isolation and loneliness with sarcopenia will not be influenced by these two factors. However, in a study by Wu, X. et al. on risk factors for sarcopenia, it was found that people living in rural areas are more likely to developing sarcopenia [40]. Conversely, Yu, B. et al. discovered that the negative impact of social isolation on physical function are more significant in males [14]. This may be because we are focusing on whether there are statistical differences in social isolation and loneliness among different places of residence, not sarcopenia. Our study also emphasizes the interaction between social isolation and loneliness.

To summarize, social isolation and loneliness have a significant impact on middle-aged and elderly in China, and we need to make improvements in these two aspects. Firstly, we need to continuously improve the social health service system; secondly, community personnel should pay regular visits to the left-behind elderly to strengthen the care for the left-behind elderly; furthermore, children’s care is very important; lastly, the society should form a good atmosphere of fraternity and love and be sufficiently tolerant to the middle-aged and elderly population. Finally, the society should form a good and friendly atmosphere and be tolerant enough to the middle-aged and elderly.

Limitations exist in our study. First and foremost, the mechanism of the synergistic effect of social isolation and loneliness is unknown, and the impact on sarcopenia is not simply the result of the combination of social isolation and loneliness. Secondly, the assessment of loneliness only reported whether loneliness was experienced in the past week. Although this single-item measurement is highly correlated with multi-item loneliness scales and has been widely used in previous studies, this measurement method may not be as reliable as using a composite measurement of multiple aspects of loneliness [17, 18]. Furthermore, the measurement of social isolation in the baseline data was based on participants’ self-narratives, which could be subject to recall bias. The recall bias of older adults may underestimate social behavioral participation. Lastly, there may be other unmeasurable factors leading to the associations found here. Therefore, no definitive causal conclusions can be drawn.

## Conclusion

Social isolation and loneliness further increase the risk of sarcopenia, and there is a synergistic effect of social isolation and loneliness. This provides new ideas and directions to improve the development of sarcopenia.

### Electronic supplementary material

Below is the link to the electronic supplementary material.

Supplementary Material 1

## Data Availability

The data used in this study are available on China Health and Retirement Longitudinal Study website: https://charls.pku.edu.cn/gy/gyxm.htm.
