# Supplementary material for: The association of social isolation and loneliness with sarcopenia among the middle-aged and elderly in China
Source: BMC Psychiatry. 2024 Jul 18;24:513. doi: 10.1186/s12888-024-05958-y (PMC11264577; doi:10.1186/s12888-024-05958-y)
Supplement: Supplementary file 1 — Supplementary Material 1 [file 12888_2024_5958_MOESM1_ESM.docx]

**Table S1.** Longitudinal association between social isolation and loneliness on sarcopenia incidence stratified by place of residence (N=7,970).

| Variable | Urban | Rural | *P* for interaction |
| --- | --- | --- | --- |
| **Social isolation** |  |  | 0.153 |
| No | 1.00(Ref) | 1.00(Ref) |  |
| Yes | 1.46(0.87-2.46) | 1.46(1.11-1.91) |  |
| **Loneliness** |  |  | 0.019 |
| No | 1.00(Ref) | 1.00(Ref) |  |
| Yes | 1.67(0.94-2.97) | 1.72(1.27-2.32) |  |
| **Combined effect of social isolation and loneliness** | | | 0.456 |
| Neither social isolation or loneliness | 1.00(Ref) | 1.00(Ref) |  |
| Social isolation alone | 1.59(0.86-2.95) | 0.77(0.41-1.42) |  |
| Loneliness alone | 1.89(0.93-3.84) | 1.35(0.94-1.95) |  |
| Both social isolation and loneliness | 1.85(0.79-4.36) | 2.20(1.51-3.22) |  |

Multivariable-adjusted for age, sex, place of residence, education level, smoking, drinking, systolic blood pressure, history of chronic diseases and medications.

**Table S****2.** Longitudinal association between social isolation and loneliness on sarcopenia incidence stratified by age (N=7,970).

| Variable | Age<60 | Age≥60 | *P* for interaction |
| --- | --- | --- | --- |
| **Social isolation** |  |  | <0.001 |
| No | 1.00(Ref) | 1.00(Ref) |  |
| Yes | 1.20(0.74-1.94) | 1.40(1.05-1.87) |  |
| **Loneliness** |  |  | <0.001 |
| No | 1.00(Ref) | 1.00(Ref) |  |
| Yes | 1.42(0.90-2.25) | 1.45(1.09-1.92) |  |
| **Combined effect of social isolation and loneliness** | | | <0.001 |
| Neither social isolation or loneliness | 1.00(Ref) | 1.00(Ref) |  |
| Social isolation alone | 0.85(0.39-1.89) | 1.21(0.72-2.06) |  |
| Loneliness alone | 1.20(0.69-2.09) | 1.20(0.83-1.74) |  |
| Both social isolation and loneliness | 1.70(0.92-3.11) | 1.74(1.17-2.59) |  |

Multivariable-adjusted for age, sex, place of residence, education level, smoking, drinking, systolic blood pressure, history of chronic diseases and medications.

**Table S****3.** Longitudinal association between social isolation and loneliness on sarcopenia incidence stratified by sex (N=7,970).

| Variable | Male | Female | *P* for interaction |
| --- | --- | --- | --- |
| **Social isolation** |  |  | 0.929 |
| No | 1.00(Ref) | 1.00(Ref) |  |
| Yes | 1.79(1.21-2.65) | 1.15(0.84-1.57) |  |
| **Loneliness** |  |  | 0.457 |
| No | 1.00(Ref) | 1.00(Ref) |  |
| Yes | 1.97(1.31-2.96) | 1.12(0.81-1.55) |  |
| **Combined effect of social isolation and loneliness** | | | 0.670 |
| Neither social isolation or loneliness | 1.00(Ref) | 1.00(Ref) |  |
| Social isolation alone | 1.35(0.64-2.83) | 0.99(0.58-1.69) |  |
| Loneliness alone | 1.70(1.04-2.79) | 1.02(0.69-1.52) |  |
| Both social isolation and loneliness | 3.13(1.84-5.32) | 1.27(0.83-1.94) |  |

Multivariable-adjusted for age, sex, place of residence, education level, smoking, drinking, systolic blood pressure, history of chronic diseases and medications.

**Table** **S4.** Longitudinal association between social isolation and loneliness on sarcopenia incidence stratified by social support (N=7,970).

| Variable | With social support | Without social support | *P* for interaction |
| --- | --- | --- | --- |
| **Social isolation** | | | <0.001 |
| No | 1.00(Ref) | 1.00(Ref) |  |
| Yes | 1.32(0.95-1.85) | 1.39(1.05-1.99) |  |
| **Loneliness** | | | <0.001 |
| No | 1.00(Ref) | 1.00(Ref) |  |
| Yes | 1.03(0.73-1.44) | 2.21(1.50-3.24) |  |
| **Combined effect of social isolation and loneliness** | | | <0.001 |
| Neither social isolation or loneliness | 1.00(Ref) | 1.00(Ref) |  |
| Social isolation alone | 0.89(0.59-1.33) | 1.10(0.55-2.18) |  |
| Loneliness alone | 1.05(0.60-1.84) | 2.01(1.24-3.24) |  |
| Both social isolation and loneliness | 1.37(0.88-2.14) | 2.64(1.61-4.32) |  |

Multivariable-adjusted for age, sex, place of residence, education level, smoking, drinking, systolic blood pressure, history of chronic diseases and medications.

**Table S****5.** Sensitivity analysis of longitudinal association between social isolation and loneliness on sarcopenia incidence (N=7,970).

| Variable | Case, n (%) | Model 1 | Model 2 | Model 3 |
| --- | --- | --- | --- | --- |
| **Social isolation** |  |  |  |  |
| No | 516(11.78) | 1.00(Ref) | 1.00(Ref) | 1.00(Ref) |
| Yes | 231(15.55) | 1.38(1.17-1.63) | 1.34(1.13-1.59) | 1.37(1.20-1.56) |
| **Loneliness** |  |  |  |  |
| No | 369(11.30) | 1.00(Ref) | 1.00(Ref) | 1.00(Ref) |
| Yes | 378(14.53) | 1.33(1.14-1.56) | 1.34(1.15-1.57) | 1.18(1.01-1.39) |
| **Combined effect of social isolation and loneliness** | | | | |
| Neither social isolation or loneliness | 280(10.71) | 1.00(Ref) | 1.00(Ref) | 1.00(Ref) |
| Social isolation alone | 236(13.35) | 1.28(1.07-1.55) | 1.30(1.08-1.56) | 1.05(0.90-1.22) |
| Loneliness alone | 89(13.65) | 1.32(1.02-1.70) | 1.28(0.99-1.65) | 1.32(1.10-1.57) |
| Both social isolation and loneliness | 142(17.03) | 1.71(1.37-2.13) | 1.68(1.35-2.09) | 1.51(1.25-1.82) |

In sensitivity analysis, those with possible sarcopenia were divided into sarcopenia.

Model 1 was unadjusted.

Model 2 was adjusted for age and sex.

Model 3 was adjusted for adjusted for age, sex, place of residence, education level, smoking, drinking, systolic blood pressure, history of chronic diseases and medications.
